# Physical assault in the previous year and total and cause-specific mortality in Russia: a case–control study of men aged 25–54 years

**DOI:** 10.1093/ije/dyw301

**Published:** 2016-12-19

**Authors:** Vishal Bhavsar, Sarah Cook, Lyudmila Saburova, David A Leon

**Affiliations:** 1 Institute of Psychiatry, King’s College, London, UK; 2 Faculty of Epidemiology and Population Health, London School of Hygiene & Tropical Medicine, London, UK; 3 Institute of Philosophy and Law, Russian Academy of Sciences (Ural branch), Ekaterinburg, Russia; 4 Arctic University of Norway, UiT, Tromsø, Norway

**Keywords:** Assault, violence, alcohol, mortality, Russia

## Abstract

**Background:** Violence has important health effects. The results of exposure to physical violence include, but may not be limited to, death from suicide and homicide. The connection between the experience of assault and risk of death from causes other than homicide and suicide has rarely been examined.

**Methods:** We analysed data from the first Izhevsk Family Study (IFS-1), a population-based case–control study of premature mortality in Russian men. Structural equation models were used to obtain odds ratios (ORs) for the association between the proxy report of physical attack in the previous year and mortality.

**Results:** The estimate of the all-cause mortality OR for assault, after adjusting for alcohol use and socio-demographic confounders, was 1.96 (95% confidence interval: 1.71, 3.31). Strong cause-specific associations were found for external causes, but associations were also found for deaths from cardiovascular and alcohol-related deaths.

**Conclusions:** We found that, in our population of working-aged Russian men, there was a strong association between physical assault and mortality from a wide range of causes. Other than direct effects of physical assault on mortality, residual confounding is an important possibility. The association between assault and mortality, particularly from cardiovascular and alcohol-related causes requires replication and further investigation.

Key Messages

- Very few epidemiological studies have assessed the relationship between physical assault and mortality from non-external causes.
- There was a strong association between all-cause mortality and proxy reports of physical assault in the last year in our population of working-aged Russian men.
- Alcohol and socio-demographic variables explained this association to some extent, but not completely.
- Assault may increase mortality from alcohol-related and cardiovascular causes but this requires further study.

## Introduction

Violence disproportionately affects the vulnerable and has universal ethical, legal, economic and health consequences.^1^ In 2010, WHO estimated there were 468,000 homicides worldwide.^2^ Violent causes rank seventeenth among the 30 most prevalent causes of death, and affect males and females in a ratio of roughly 2:1.^3^ Non-lethal violence data are sparser, derived in different ways by country, and may be unreliable. Estimates for such violence have focused on particular contexts: 10–69% of women report intimate partner violence; 20% of women and 5–10% of men report experience of childhood sexual abuse; and 4–6% of older people report violence.^4^

In the decade following the collapse of the Soviet Union, there was an increase in reported crime in a number of post-Communist states.^5^ In the mid-1990s, homicide rates in Russia were 15–20 times greater than in most European countries^6^; in 2004, there were 22.1 homicides per 100,000 population and 40.1 cases of grievous bodily harm.^7^ There is also some evidence that non-lethal violence has not been uncommon in Russia. In a 2004 Moscow population survey of 1190 men and women aged over 18, the 12-month prevalence of assault victimization was 8.7% overall, and 12.5% in men.^8^

Relatively consistent positive associations between assault and characteristics such as younger age, male gender, lower socio-economic position, single marital status and alcohol misuse have been found in crime surveys from Anglophone countries,^9–12^ but evidence on Russia is limited. In the Moscow study discussed above, age-adjusted associations with assault were found for binge drinking, social network-capital, but not income, education or marital status.^8^ Stickley *et al.*^13^ report an analysis of the association of assault with health in nine former Soviet countries. The 12-month prevalence of assault in Russia was 1.3%. In a fully adjusted regression model, associations were found for the risk of assault with gender, age and alcohol, but also with being never married, and not socializing with neighbours.

Violence is associated with a range of adverse health effects.^14–16^ Intimate partner violence (IPV) is associated with suicide attempts in women^17^^,^^18^ and there is some evidence linking non-fatal IPV to later intimate partner homicide.^19^ Increasing evidence indicates important health consequences of non-lethal IPV, e.g. injury, chronic pain and depression.^20^ In Russia, Stickley *et al.*^13^ found those who were physically assaulted were at 2.5 times the odds of self-reporting poor health and 2.9 times the odds of reporting poor psychological health compared with those who were not.

Even since the sharp decline in male life expectancy in Russia in the 1990s, and then the improvements seen since the mid-2000s, premature mortality has remained very high compared with that seen in European countries.^21^ Rates of mortality by suicide and homicide among working-aged Russian males are among the highest in Europe,^22^^,^^23^ suggesting that there may be a large iceberg of non-fatal violence underlying these deaths. However, the connection between the experience of assault and subsequent risk of death from causes other than homicide and suicide has rarely been examined in any country. The potential mechanisms that may link assault with mortality from non-violent causes are several. In the Russian context, hazardous alcohol drinking may play an important role, either as a common cause (of the assault and subsequent death) or as a response to assault. Certainly, hazardous drinking in Russia has been associated with elevated mortality from a wide range of causes, particularly in men.^24^^,^^25^

We have used the opportunity afforded by a unique case–control study conducted in the city of Izhevsk in Russia 2003–05 to investigate these issues further. Whereas the mortality and alcohol-consumption patterns of Russian men are rather exceptional, as the literature is so sparse, this setting provides a good basis for investigating the links between assault and mortality, and the potential role of alcohol, which will be of general interest to researchers and policy-makers.

## Methods

We analysed data from the first Izhevsk Family Study (IFS-1), a population-based case–control study of premature mortality in Russian men. The study protocol is described in detail elsewhere.^24^ Izhevsk is a demographically typical industrial city in the Western Urals, Russia, with a similar average life expectancy as for Russia as a whole, and a similar distribution of deaths by cause in working-aged men. Cases were 1750 male Izhevsk residents who died from any cause aged 25–54 years (2003–05) and who resided in households with at least one other person at the time of death. Cases were notified directly to the study team by the registrar of deaths (ZAGS). Cause of death was classified according to ICD-10 by the certifying doctors/pathologists. About a third of deaths were attributed to external causes (injuries, poisonings and violence, *n* = 544) and a third to cardiovascular disease (*n* = 573). In this analysis, we use 1750 controls who were live men randomly selected from a 2002 population register and frequency-matched within 5-year age bands to cases notified for that month; in addition, we use an additional 250 controls who were recruited in 2006 using the same protocol as used for the initial case–control study. The reason for this was to increase the sample size for a planned follow-up study of the controls.

Interviews of controls took place between 2003 and 2006. Trained interviewers obtained information about the lives and behaviours of cases and controls from proxy respondents living in the same household. Proxies were mainly wives, mothers or daughters. For the cases, proxy interviews occurred around 2 months after the death. Information was collected on socio-demographics, health behaviours, violence and physical health. The primary exposure in this study (physical assault) was determined according to the proxy response to a question about whether the subject had been physically assaulted in the past year. The text of this question in Russian was ‘Жертвой каких из перечисленных ниже преступлений становился *этот человек* в течение последнего года?: ему были нанесены телесные повреждения любым способом’ (‘I now want to ask you which, if any, of the following crimes were committed against *the subject* during the past year: someone physically assaulted him’). For the controls, interviews were obtained from both the control and a proxy for 1941 men, but, for the main analyses, the data reported by the controls have not been used. Proxy information from controls as well as cases was used, in order to minimize differential measurement error in case and control information. Previous analyses of the relationship between control and proxy responses found moderate agreement of measures of alcohol use and near perfect agreement for markers of socio-economic position.^26^^,^^27^ The prevalence of assault as reported by the controls themselves was 86/1941 (4.4%). This compares with 4.7% from the proxy reports. The kappa statistic for inter-rater agreement between proxies and controls was 0.51 (suggesting moderate agreement). Interviewers were instructed to interpret the item measuring assault in men who died of homicide as it related to assaults not including the incident leading to death. However, the overlap between homicide and reported assault may have introduced measurement error for the exposure and, to this extent, may result in overestimation of strength of association of history of assault prior to the circumstances associated with a homicide. Interviewers were blinded as to the cause of death for cases; however, a question later in the questionnaire was included that asked the proxy to give their opinion as to the cause of death with the options disease/homicide/suicide/accidental injury/poisoning by alcohol or other spirits/accidental cause (including drowning).

### Potential confounding variables

Alcohol use was considered a priori as an important confounder or common cause of the association between physical assault and mortality. Given the multi-dimensional nature of alcohol use and the generally poor reliability of obtaining accurate measurement of alcohol volume from proxy reports, we used a series of previously developed alternative measures aimed at identifying aspects of heavy drinking that would be readily observable by a proxy informant who lived in the same household as a study subject.^28^ Information was gathered on the frequency of episodes of *zapoi* (periods of continuous drunkenness with withdrawal from normal social life for two or more days) and on the consumption of non-beverage alcohols. Non-beverage alcohols are manufactured ethanol-containing substances not intended for drinking. These dimensions of hazardous drinking behaviour have been shown previously to be strongly associated with mortality in this population.^24^

In the analysis, we used a measure of acute alcohol-related dysfunction in the form of a latent variable manifested by the frequencies of four types of alcohol-related behaviour: hangover, excessive drunkenness, sleeping in clothes because of drunkenness and failing personal or family obligations because of drinking alcohol (see Supplementary Data, available at *IJE* online). This measure has been previously described and published based on the present data.^29^^,^^30^ Sporadic alcohol dysfunction was measured by the frequency of *zapoi* in the past year (never, sometimes, ever). The drinking of surrogate or non-beverage alcohols was measured using a binary variable for use in the past 12 months.

Other potential confounding variables included were smoking, employment status at time of interview, educational attainment, lifetime history of imprisonment and adverse life events (including serious illness of wife/partner, serious illness of other close friend/family, death of wife/partner, death of other close friend/family, divorce/separation, serious financial problems, other serious problems involving family/friends and serious employment problems). Socio-economic position was measured using an amenities index derived from whether the man’s household had a car and central heating categorized as neither, one or both.

Maximum likelihood estimation was used to obtain odds ratios (ORs) for the association between physical attack and mortality estimated from structural equation models. Separate models were fitted including (i) age only, (ii) age and all potential confounders except alcohol use, (iii) age and alcohol use and (iv) all potential confounders. Models which adjusted for alcohol use also included a measurement component consisting of the latent variable acute alcohol-related dysfunction.^29^

Separate models were fitted for all-cause mortality and for specific causes, based on chapters of the ICD-10.^31^ Explicitly alcohol-related deaths were put in a separate category defined by combining deaths from alcohol-related mental disorders, alcoholic cardiomyopathy, alcoholic liver disease and acute alcohol poisoning. External causes were further sub-divided into case sets for homicide and suicide. Deaths from suicide were analysed separately and also jointly with injury/violent deaths of undetermined intent.

Model fit for the measurement model was assessed using the confirmatory fit analysis (CFI), the Tucker Lewis Index (TLI) and the Root Mean Square Error of Approximation (RMSEA). CFI and TLI values greater than 0.95 indicate good model fit, with a minimum of 0.90 indicating acceptable fit.^32^^,^^33^ For the RMSEA, values greater than 0.1 indicate a bad fit, whereas less than 0.08 indicates a reasonable fit and values less than 0.05 indicate a good fit. For the structural equation models, data missing due to item non-response for the observed indicators of the latent factor were estimated via the Expectation Maximization (EM) algorithm. This method is valid under the assumption that data are missing at random. For the other covariates, incomplete records were excluded.

The roles of smoking and socio-economic status as confounders of the association between physical assault and mortality were investigated further in supplementary analyses. Structural equation models were fitted to look at the association between smoking and mortality by specific causes and smoking and physical assault in controls after adjusting for all other potential confounders including alcohol use. The roles of socio-economic variables and smoking as confounders of the association between assault and mortality were assessed by fitting model (ii) as stated above but with and without adjustment separately for (i) smoking and (ii) socio-economic status.

The adjusted population attributable fraction and corresponding confidence intervals were estimated according to formulae from Greenland.^34^ All analyses were done in Stata version 14^35^ and Mplus version 7.^36^

Oral consent was obtained from proxy informants. Ethical approval for the study was obtained from the committees of the Izhevsk Medical Academy and the London School of Hygiene and Tropical Medicine. Further information about the Izhevsk Family Study and information about how to apply for the data for research are available at http://www.ifsmetadata.info.

### Role of the funding source

The corresponding author had full access to all anonymized data in the study and had final responsibility for the decision to submit for publication.

## Results

The study comprised 1750 cases and 2000 controls. Counts of observations and missing data by case–control status for each covariate are displayed in Table 1, along with the age-adjusted association of each covariate with having being assaulted in the previous year. In order to understand the association of a history of assault with a range of behavioural and socio-demographic factors in the target study population of the city of Izhevsk, we first analysed the control proxy data on their own. All the alcohol and socio-demographic covariates showed strong associations with assault.

**Table 1.** Characteristics of cases and controls in the Izhevsk Family Study as reported by proxy informants and association of covariates with risk of assault in controls

	Controls (%) (live men)	Cases (%) (dead men)	Age-adjusted OR for assault in controls
**Assault in past year**			
No	1906 (95.3)	1463 (83.6)	
Yes	94 (4.70)	287 (16.4)	
**Age (years)**			
25–29	144 (7.2)	131 (7.5)	
30–34	163 (8.2)	144 (8.2)	
35–39	171 (8.6)	136 (7.8)	
40–44	336 (16.8)	306 (17.5)	
45–49	491 (24.6)	441 (25.2)	
≥50	695 (34.8)	592 (33.8)	
**Non-beverage alcohol use ever**			
No	1813 (90.7)	988 (56.5)	1 (reference)
Yes	159 (8)	1717 (41)	3.35 (1.93, 5.81)
Missing	28 (1.4)	45 (2.6)	
Total	1972 (100)	1705 (100)	
**Frequency of being excessively drunk**			
Never or almost never	826 (41.3)	440 (25.14)	1 (reference)
Less than once a month	406 (20.3)	192 (10.97)	1.22 (0.65, 2.29)
Once a month	226 (11.3)	179 (10.23)	1.99 (1.02, 3.87)
Several times a month	105 (5.25)	181 (10.34)	2.91 (1.32, 6.42)
Once a week	79 (3.95)	108 (6.17)	2.61 (1.04, 6.57)
Several times a week	65 (3.25)	274 (15.66)	6.85 (3.18, 14.76)
Every day	24 (1.2)	200 (11.43)	4.79 (1.33, 17.27)
Missing	269 (13.45)	176 (10.06)	
**Frequency of hangover**			
Never or almost never	904 (45.2)	497 (28.4)	1 (reference)
Less than once a month	340 (17)	163 (9.3)	1.43 (0.76, 2.67)
Once a month	206 (10.3)	140 (8)	1.8 (0.90, 3.59)
Several times a month	110 (5.5)	157 (8.97)	3.61 (1.78, 7.31)
Once a week	60 (3)	91 (5.2)	1.56 (0.46, 5.29)
Several times a week	53 (2.65)	263 (15.03)	5.58 (2.39, 13.04)
Every day	24 (1.2)	222 (12.69)	8.35 (2.88, 24.20)
Missing	303 (15.15)	217 (12.4)	
**Frequency of failing family or personal obligations because of drinking**			
Never or almost never	1272 (63.6)	792 (45.26)	1 (reference)
Less than once a month	135 (6.75)	94 (5.37)	1.38 (0.57, 2.88)
Once a month	122 (6.1)	81 (4.63)	1.98 (0.9, 3.89)
Several times a month	82 (4.1)	103 (5.89)	1.22 (0.42, 3.41)
Once a week	48 (2.4)	57 (3.26)	2.32 (0.74, 6.16)
Several times a week	47 (2.35)	220 (12.57)	5.36 (2.14, 10.82)
Every day	20 (1)	172 (9.83)	4.91 (1.37, 17.57)
Missing	274 (13.7)	231 (13.2)	
**Frequency of sleeping in clothes because of drunkenness**			
Never or almost never	1317 (65.85)	742 (42.4)	1 (reference)
Less than once a month	162 (8.1)	111 (6.34)	1.38 (0.64, 2.97)
Once a month	114 (5.7)	101 (5.77)	1.19 (0.45, 2.99)
Several times a month	60 (3)	145 (8.29)	5.97 (2.81, 12.71)
Once a week	28 (1.4)	77 (4.4)	2.13 (0.49, 9.34)
Several times a week	55 (2.75)	253 (14.46)	6.64 (3.11, 14.15)
Every day	13 (0.65)	150 (8.57)	9.97 (2.59, 38.37)
Missing	251 (12.55)	171 (9.77)	
**Frequency of *zapoi***			
Never	1546 (77.3)	889 (50.8)	1 (reference)
Sometimes	14 (7.1)	260 (14.9)	1.69 (0.82, 3.49)
Often	64 (3.2)	441 (25.2)	6.51 (3.33, 12.74)
Missing	249 (12.5)	160 (9.1)	
**Smoking**			
Never smoked	421 (21.1)	133 (7.6)	1 (reference)
Ex-smoker	252 (12.6)	149 (8.5)	1.21 (0.42, 3.44)
1–10/day current	468 (23.4)	464 (26.5)	1.92 (0.86, 4.3)
11–20/day current	656 (32.8)	710 (40.6)	2.96 (1.42, 6.19)
>20/day current	201 (10.1)	294 (16.8)	5.68 (2.51, 12.84)
Missing	2 (0.1)	0 (0)	
**Employment**			
Yes	1665 (83.3)	693 (39.6)	1 (reference)
No	332 (16.6)	1056 (60.3)	2.46 (1.98, 3.06)
Missing	3 (0.2)	1 (0.1)	
**Car/central heating**			
Both	750 (37.5)	333 (19)	1 (reference)
Only one	1094 (54.7)	1173 (67)	1.82 (1.38, 2.4)
Neither	156 (7.8)	244 (13.9)	2.23 (1.53, 3.27)
**Education**			
Higher	438 (21.9)	171 (9.7)	1 (reference)
Secondary	1437 (71.9)	1342 (76.7)	1.46 (1.05, 2.01)
Incomplete secondary	108 (5.4)	209 (11.9)	1.93 (1.23, 3.04)
Missing	17 (0.9)	28 (1.6)	
**Ever been imprisoned**			
No	1904 (95.2)	1449 (82.8)	1 (reference)
Yes	91 (4.6)	292 (16.7)	2.88 (1.47, 5.68)
Missing	5 (0.3)	9 (0.5)	
**Any adverse life event in past year**			
No	1044 (52.2)	817 (46.7)	1 (reference)
Yes	954 (47.7)	931 (53.2)	2.01 (1.3, 3.09)
Missing	2 (0.1)	2 (0.1)	
**Marital status**			
Cohabiting, married	1544 (77.2)	930 (53.1)	1 (reference)
Cohabiting, unmarried	201 (10.1)	205 (11.7)	1.9 (1.05, 3.46)
Divorced	120 (6)	342 (19.5)	1.95 (0.94, 4.06)
Widowed	17 (0.9)	57 (3.3)	–
Never married	118 (5.9)	215 (12.3)	2.536 (1.15, 4.85)
Missing	0 (0)	1 (0.1)	
Total	2000 (100)	1750 (100)	

The associations of mortality by cause with assault are shown in Table 2. The age-adjusted all-cause mortality odds ratio for assault was more than 4. This was, however, attenuated by the addition of non-alcohol-related covariates. The inclusion of just the alcohol-related covariates resulted in a more substantial attenuation and addition of all covariates produced a fully adjusted estimate of the odds ratio for assault of just under 2.

**Table 2.** Regression models for the effect of physical assault on specific and combined causes of mortality

		Model I*			Model II*			Model III*			Model IV*		
	Number of cases	Odds ratio	Lower 95% CI	Upper 95% CI	Odds ratio	Lower 95% CI	Upper 95% CI	Odds ratio	Lower 95% CI	Upper 95% CI	Odds ratio	Lower 95% CI	Upper 95% CI
**Chapter-level causes (ICD-10 codes)**													
Infections and parasitic diseases (I)	**53**	**3.02**	0.93	9.78	**1.29**	0.29	5.77	**1.33**	0.36	5.01	**0.99**	0.20	4.82
Neoplasms (II)	**170**	**2.07**	0.86	4.99	**1.58**	0.59	4.24	**1.66**	0.67	4.12	**1.63**	0.60	4.43
Mental and behavioural disorders (V)	**19**	**7.10**	1.77	28.5	**3.02**	0.60	15.31	**2.22**	0.42	11.76	**1.75**	0.27	11.47
Diseases of circulatory system (IX)	**573**	**3.51**	2.23	5.52	**2.09**	1.25	3.51	**2.07**	1.24	3.46	**1.78**	1.03	3.07
Diseases of respiratory system (X)	**137**	**3.29**	1.58	6.91	**1.67**	0.68	4.09	**1.38**	0.56	3.36	**1.22**	0.46	3.21
Diseases of digestive system (XI)	**182**	**4.47**	2.45	8.15	**2.76**	1.37	5.55	**1.78**	0.83	3.78	**1.80**	0.81	4.01
External causes (XX)	**544**	**5.62**	3.77	8.38	**3.82**	2.43	6.00	**3.39**	2.12	5.40	**3.12**	1.92	5.05
Other	**72**	**3.91**	1.51	10.1	**2.20**	6.77	0.72	**2.00**	0.68	5.84	**1.89**	0.58	6.16
*All causes of death*	***1750***	***4.15***	*2.96*	*5.81*	***2.92***	*2.00*	*4.26*	***2.42***	*1.63*	*3.59*	***1.96***	*1.71*	*3.31*
**Selected causes**													
Circulatory disease**:													
Ischaemic heart disease (I20–25)	**258**	**2.14**	1.05	4.34	**1.30**	0.60	2.85	**1.52**	0.71	3.24	**1.21**	0.54	2.70
Other Cardiomyopathy (I42, except I42.6)	**61**	**1.90**	0.54	6.63	**0.88**	0.22	3.45	**0.93**	0.24	3.63	**0.66**	0.16	2.81
Cerebrovascular disease (I60–69)	**100**	**4.36**	1.90	10.00	**2.92**	1.15	7.41	**3.08**	1.27	7.46	**2.87**	1.10	7.50
Other circulatory disease (I00–I99, except I20–25, I42 and I60–69)	**33**	**2.30**	0.46	11.51	**1.03**	0.17	6.04	**1.68**	0.31	9.16	**1.07**	0.18	6.48
**Alcohol-related**													
Mental disorders due to alcohol (F10)	**18**	**7.69**	1.89	31.25	**3.12**	0.61	16.04	**2.37**	0.43	13.01	**1.81**	0.27	12.04
Alcoholic cardiomyopathy (I42.6)	**121**	**6.80**	3.59	12.85	**3.74**	1.77	7.91	**2.87**	1.25	6.57	**2.45**	1.03	5.83
Alcoholic liver disease (K70)	**74**	**7.58**	3.54	16.23	**4.65**	1.84	11.75	**2.69**	1.02	7.12	**2.76**	0.92	8.31
Acute alcohol poisoning (X45)	**95**	**5.64**	2.73	11.64	**3.57**	1.58	8.09	**2.44**	1.01	5.92	**2.06**	0.81	5.25
*Overall*	**308**	***6.55***	*4.14*	*10.36*	***4.19***	*2.42*	*7.26*	***2.82***	*1.52*	*5.26*	***2.65***	*1.38*	*5.08*
External causes***													
Transport injuries (V01–V99)	**42**	**4.61**	1.57	13.60	**4.18**	1.27	13.74	**4.79**	1.52	15.14	**4.97**	1.39	17.72
Other accidental poisoning (X40–X49 except X45)	**33**	**3.70**	1.11	12.36	**1.47**	0.35	6.12	**1.92**	0.51	7.31	**1.12**	0.25	5.03
Drowning (W65–W74)	**18**	**1.07**	0.07	15.65	**0.46**	0.03	7.89	**0.27**	0.01	5.1	**0.25**	0.01	5.57
Exposure to cold (X31)	**29**	**10.04**	3.28	30.76	**5.15**	1.48	17.95	**4.43**	1.25	15.67	**3.7**	0.96	14.22
Other accidental deaths (V0–X59, no including V01–V99, X40–49, W65–W74 and X31)	**35**	**3.67**	1.00	13.49	**1.83**	0.42	8.01	**1.34**	0.3	6.06	**1.2**	0.22	6.44
Suicide (X60–X84)	**120**	**5.35**	2.82	10.16	**3.36**	1.64	6.89	**2.96**	1.44	6.09	**2.7**	1.28	5.7
Homicide (X85–Y09)	**45**	**11.82**	4.62	30.25	**7.68**	2.6	22.71	**5.93**	2.07	17.00	**6.63**	2.14	20.59
Undetermined intent (Y10–Y34)	**111**	**6.70**	3.46	12.95	**4.39**	2.07	9.32	**3.43**	1.59	7.38	**3.3**	1.46	7.49
All other external causes	**16**	**1.20**	0.08	18.09	**0.87**	0.05	15.91	**0.58**	0.03	10.61	**0.55**	0.03	12.26
Undetermined intent plus suicide****	**231**	**5.99**	3.62	9.92	**2.28**	4.03	7.13	**3.39**	1.90	6.07	**3.22**	1.75	5.9
All causes except external causes	**1206**	**3.58**	2.46	5.21	**2.32**	1.51	3.58	**1.97**	1.28	3.02	**1.89**	1.19	2.99

* Model I: adjusted for Model I: adjusted only for age, Model II: Model 1 + adjusted for smoking, employment, car/central heating ownership, education, imprisonment, any adverse life event, marital status; Model III: Model II plus alcohol-related dysfunction [*zapoi*, surrogates, and acute alcohol-related dysfunction (latent)]; Model IV: Adjusted for all variables.

** Circulatory disease also included deaths from alcoholic cardiomyopathy, which is presented with ‘Constituting deaths from alcohol’.

*** External causes also included acute alcohol poisoning, which is presented with ‘Constituting deaths from alcohol’.

**** Based on the same data as estimates for ‘suicide’ and ‘undetermined’.

95% CI, 95% confidence interval.

The association with external causes was particularly strong, but also displayed substantial attenuation on adjusting for socio-demographic and alcohol covariates. Among external causes, after adjusting for full model covariates, mortality from transport injuries, suicide (including when combined with undetermined causes), homicide and deaths from undetermined cause showed good evidence of an association with the physical assault.

Among non-external causes, good evidence for associations remained after adjustment for all potential confounding variables factors for circulatory disease only; after adjustment, people who had been assaulted were nearly two times more likely to die from circulatory disease compared with those who were not assaulted. Among specific circulatory causes, fully adjusted ORs for both alcoholic cardiomyopathy and cerebrovascular disease were more than 2. Assault remained associated with death from digestive causes after adjusting for socio-demographic variables, but this association became too imprecise upon addition of alcohol-related variables to the model, although the point estimate remained more than 2.

There was a strong association between alcohol-related causes of death and physical assault with an odds ratio of 4.2 after adjustment for confounders other than alcohol. For individual causes, there was strong evidence of an association for alcoholic cardiomyopathy, alcoholic liver disease and alcohol poisoning. As expected, the strength of the association reduced on adjusting for alcohol use but there remained good evidence for an association between physical assault and all alcohol-related causes of death and for alcoholic cardiomyopathy even after adjustment for alcohol use.

The results of further investigation of the role of smoking and socio-economic status as confounders of the association between physical assault and mortality are shown in the Supplementary Data (Supplementary Tables 1–4, available as Supplementary data at *IJE* online). Although smoking was a strong risk factor for mortality including mortality from external causes (Supplementary Table 1, available as Supplementary data at *IJE* online), the strong association between smoking and physical assault displayed in Table 1 was much attenuated on adjustment for other variables including alcohol (Supplementary Table 2, available as Supplementary data at *IJE* online). Leaving out adjustment for smoking from the models had only a small effect on effect estimates for the association between assault and mortality, and did not change our substantive conclusions (Supplementary Table 3, available as Supplementary data at *IJE* online). The same was true for models with and without adjustment for socio-economic status (Supplementary Table 4, available as Supplementary data at *IJE* online).

The adjusted population attributable fraction for mortality related to assault was 8.69% (95% confidence interval: 8.3, 9.3).

## Discussion

To our knowledge, this is the first study to investigate the link between physical assault and mortality. We found that, in our population of working-aged Russian men, there was a strong association between physical assault and mortality from a wide range of causes. These effects were only partially removed on adjustment for alcohol and socio-demographic factors. The largest effects were seen for external causes, followed by circulatory diseases, especially stroke. Nevertheless, the attenuation of effects seen on adjustment was substantial in many instances, as would be expected from the very strong associations seen in Table 1 between assault and the potential confounders.

There are a number of potential explanations for our results. First, there could be a direct effect of assault on mortality. Although we found an association with homicide deaths as anticipated, associations were also found for suicides, alcohol-related causes and cardiovascular mortality, indicating that not all the mortality burden of assault is carried by direct injuries. The strong and persistent association with deaths from transport injuries is less easy to understand.

Second, although the association between assault and all-cause premature male mortality is consistent with causal pathways, assaulted people are likely to have differed from the rest of the population in important ways. The measure of assault could be an indicator of violent, risky, hazardous lifestyles, which in turn could be associated with mortality. In particular, assault and premature male mortality might share a common root cause, such as alcohol or deprivation, which was not adequately accounted for in this study. For example, it could be that people who are heavy drinkers have a pattern of behaviour and interactions with others that simultaneously put them at increased risk of assault and of death from alcohol-related causes, as well as other causes such as stroke. Alcohol misuse was prevalent among subjects (65% of cases and 45% of controls had been excessively drunk in the past 12 months) and was a major potential confounder for this hypothesis. Considerable attention was given to measuring alcohol use in detail, by assessing markers of alcohol-related dysfunction as a latent variable, the frequency of *zapoi* and the ingestion of non-beverage alcohols. However, the fact that the associations with the aggregate of alcohol-related causes remained even after adjustment for alcohol use in the past year suggests that there might be residual confounding by alcohol use, despite the density of information on various dimensions of alcohol ingestion we obtained from proxies. From this point of view, the experience of physical assault may be an additional indicator, not captured through the detailed questions on level and pattern of drinking, of hazardous drinkers at a certain stage of their drinking careers who are at risk of harm, both through behaviour that may increase their risk of being assaulted and through damage to their health physically through the biological effects of alcohol. Alternatively, it could be that assault, severe psychosocial disturbance with heavy alcohol use and physical disease all result from behavioural/lifestyle aspects of poverty not adequately accounted for here, and that there is residual confounding through socio-economic circumstances. However, it is worth noting that adjustment for socio-economic variables measured in this study did not have a strong impact on the size of the effect estimates.

A third explanation is that assault could lead to increased alcohol consumption and this could lead to death; the association of assault with deaths from alcohol-related causes is consistent with alcohol ingestion as a coping strategy following assault, given the information-processing changes that ensue.^37^ Although alcohol was adjusted for in final models, if alcohol use is an intermediate factor in a causal relationship between assault and mortality, the true magnitude of the association will have been under-estimated.

Lastly, assault could result in premature mortality via neuroendocrine stress pathways, or alterations in other behaviours that result in increased risk of premature death.^38^^,^^39^ For example, whereas the association with cardiovascular deaths is difficult to explain in terms of direct physical effects of assault, such an association does fit into a model where victimization results in changes in stress-responsiveness and changes in health- and safety-related behaviours.^40–43^ In this respect, our results are consistent with prior beliefs and with literature describing widespread health effects of violence.^13^^,^^44^

Overall, our analyses are unable to discriminate between these possibilities; direct effects, indirect effects and the many confounding-related explanations could each account for the associations reported here. However, whereas the exact mechanism for this is unclear, we have identified the experience of physical assault as a marker of increased risk of mortality, which is of importance in itself, particularly in Russia—a country with high premature mortality.

### Limitations and strengths

The design of the study necessarily meant that all data on the physical assault and potential confounding factors were collected from proxy respondents. Proxy information from controls as well as cases was used. In a previous analysis, very good agreement was found between control and control proxy data for socio-economic variables.^27^ Proxies of cases might have been more likely than proxies of controls to remember events because the man then died, which could have resulted in a bias of the association away from null. Whereas a plausible mechanism for differential exposure misclassification exists, prevalence of reported assault was similar in information collected from the controls themselves to when collected from their proxy respondent (4.4% and 4.7%, respectively), suggesting reasonable correspondence between case–control and proxy reports. Nevertheless, the proxy information must inevitably involve some element of misclassification.

This was primarily a complete records analysis. Like all analyses with missing data, mechanisms for missing data and the influence of these missing data on the results cannot be directly estimated. Although we adjusted for a range of important confounders, the possibility of residual confounding or confounding by unknown factors remains. Because of the need for proxy information on cases, the study population was restricted to cases and controls that resided with one or more household member. Plausibly, the effect of assault on mortality could be different by whether the person lives alone, limiting generalizability. The study was conducted between 2003 and 2006; however, given limited evidence on this association elsewhere in the literature, we consider the results interesting despite the fact that they relate to mortality over a decade ago when, in Russia, alcohol consumption was higher and mortality was also higher.

This is the first time the proxy report of physical assault in the previous year has been used as an indicator in an epidemiological study and it is likely to include a wide range of levels of assault. Our results are not able e.g. to differentiate between being simply pushed and being stabbed. Assault was operationalized as dichotomous, limiting the evaluation of whether effects on mortality varied by assault characteristics, e.g. severity/number of assaults.^45^ Furthermore, the item does not discriminate between instances of violence where the victim was also a perpetrator of violence or whether the victim knew the perpetrator. However, there is no universal method of measuring assault, because the evaluation of crimes as causal factors for health is relatively recent, and social scientific study of victims has lagged behind that of perpetrators.^46^ The measurement of violent events is also prone to misreporting. For example, intra-family violence might be under-reported by the spouse; in this regard, control and case proxies might have in some instances provided false negative for the exposure.

## Concluding remarks

Violence research involving unrestricted populations, rather than vulnerable groups, should form a larger part of public health landscape; increasing evidence points to its continued omission as an indicator of a structural inequality akin to the historic exclusion of socio-economic data in past epidemiologic research.^47^ Strategies to reduce/eliminate assault might result in mortality reductions, including from ‘internal’ causes. Just under 10% of cases of premature death in the target population might be prevented by eliminating assault, under strong assumptions of a causal relationship, complete exposure removability, the immediacy of effects of exposure removal and lack of competing effects. The suggestion that interventions on personal safety could have important effects on mortality resonates with public health debates on the integration of epidemiological designs with questions of social policy.^48^ The association between assault and cardiovascular and alcohol-related causes requires replication and further investigation.

## Supplementary Data

Supplementary data are available at *IJE* online.

## Funding

This work was supported by the Wellcome Trust, grant numbers 067232 and 078557 (www.wellcome.ac.uk/Funding). The funders had no role in the study design, data collection and analysis, decision to publish or preparation of the manuscript.

**Conflict of interest:** The authors have no conflicts of interest to declare.

## Supplementary Material

Supplementary Figure

Supplementary Table S1-S4
