# Supplementary material for: Physical assault in the previous year and total and cause-specific mortality in Russia: a case–control study of men aged 25–54 years
Source: Int J Epidemiol. 2016 Dec 19;46(3):1018–28. doi: 10.1093/ije/dyw301 (PMC5837239; doi:10.1093/ije/dyw301)
Supplement: Supplementary Figure [file supplementary_figure_dyw301.doc]

**Supplementary Material: Measurement model for routine alcohol-related dysfunction, based on 3236 subjects**

0.99(0.97,1)

0.98 (0.96,1)

1(constrained)

Frequency of hangover(proxy-report)

Frequency of excessive drunkenness(proxy-report)

Frequency of sleeping in clothes because of drunkenness (proxy-report)

Frequency of failing family or personal obligations because of drinking alcohol (proxy-report)

Model Fit indices:

CFI:0.99

TLI:0.99

RMSEA:0.037
