# Supplementary material for: Physical assault in the previous year and total and cause-specific mortality in Russia: a case–control study of men aged 25–54 years
Source: Int J Epidemiol. 2016 Dec 19;46(3):1018–28. doi: 10.1093/ije/dyw301 (PMC5837239; doi:10.1093/ije/dyw301)
Supplement: Supplementary Table S1-S4 [file supplementary_table_s1-s4_dyw301.doc]

| **Table S1: Showing estimates of the association between smoking and mortality by cause, after adjusting for all variables, including physical assault.** | | | | | | | | | | | | | | |
| --- | --- | --- | --- | --- | --- | --- | --- | --- | --- | --- | --- | --- | --- | --- |
|  | **Number of cases** | **Mortality OR for never smokers** | **Mortality OR for ex-smokers** | | | **Mortality OR for 1-10/day current smokers** | | | **Mortality OR for 11-20/day current smokers** | | | **Mortality OR for >20/day current smokers** | | |
| **Chapter-level causes (ICD-10 codes):** |  |  | **Odds Ratio** | **Lower 95% CI** | **Upper 95% CI** | **Odds Ratio** | **Lower 95% CI** | **Upper 95% CI** | **Odds Ratio** | **Lower 95% CI** | **Upper 95% CI** | **Odds Ratio** | **Lower 95% CI** | **Upper 95% CI** |
| **Infections and parasitic diseases (I)** | **53** | Reference | 1.16 | 0.3 | 4.6 | 0.47 | 0.14 | 1.67 | 0.45 | 0.13 | 1.6 | 1.1 | 0.28 | 4.28 |
| **Neoplasms (II)** | **170** | Reference | 2.48 | 1.27 | 4.83 | 1.19 | 0.62 | 2.31 | 1.68 | 0.91 | 3.11 | 2.04 | 0.99 | 4.18 |
| **Mental and behavioural disorders (V)** | **19** | Reference | 1.62 | 0.15 | 17.65 | 0.82 | 0.11 | 6.23 | 0.82 | 0.12 | 5.56 | 1.6 | 0.2 | 12.62 |
| **Diseases of circulatory system (IX)** | **573** | Reference | 1.16 | 0.73 | 1.83 | 1.58 | 1.1 | 2.27 | 1.94 | 1.38 | 2.73 | 2.4 | 1.62 | 3.55 |
| **Diseases of respiratory system (X)** | **137** | Reference | 2.16 | 0.93 | 5.05 | 1.31 | 0.61 | 2.81 | 1.13 | 0.54 | 2.39 | 1.8 | 0.79 | 4.1 |
| **Diseases of digestive system (XI)** | **182** | Reference | 2.24 | 0.94 | 5.35 | 3.47 | 1.71 | 7.01 | 2.4 | 1.2 | 4.83 | 1.43 | 0.62 | 3.28 |
| **External causes (XX)** | **544** | Reference | 0.93 | 0.55 | 1.6 | 1.48 | 1.00 | 2.17 | 1.78 | 1.24 | 2.57 | 1.86 | 1.21 | 2.87 |
| **Other** | **72** | Reference | 1 | 0.33 | 3.01 | 1.08 | 0.47 | 2.49 | 0.55 | 0.23 | 1.3 | 0.42 | 0.13 | 1.31 |
| ***All causes of death*** | ***1750*** | Reference | 1.51 | 1.11 | 2.05 | 1.93 | 1.5 | 2.47 | 2.03 | 1.6 | 2.58 | 2.24 | 1.69 | 2.98 |
| **Selected causes:** |  |  |  |  |  |  |  |  |  |  |  |  |  |  |
| **Circulatory disease**:** |  |  |  |  |  |  |  |  |  |  |  |  |  |  |
| **Ischaemic Heart Disease (I20-25)** | **258** | Reference | 1 | 0.53 | 1.9 | 1.58 | 0.96 | 2.6 | 2.04 | 1.28 | 3.26 | 2.5 | 1.48 | 4.24 |
| **Other Cardiomyopathy (I42, except I42.6)** | **61** | Reference | 1.51 | 0.41 | 5.55 | 1.65 | 0.59 | 4.61 | 2.8 | 1.06 | 7.41 | 2.14 | 0.66 | 6.94 |
| **Cerebrovascular disease (I60-69)** | **100** | Reference | 1.62 | 0.6 | 4.41 | 1.61 | 0.66 | 3.9 | 2.09 | 0.9 | 4.84 | 3.72 | 1.53 | 9.03 |
| **Other circulatory disease (I00-I99, except I20-25, I42, and I60-69)** | **33** | Reference | 0.85 | 0.18 | 4.02 | 1.28 | 0.38 | 4.28 | 1.29 | 0.4 | 4.19 | 1.07 | 0.25 | 4.63 |
| **Alcohol-related:** |  |  |  |  |  |  |  |  |  |  |  |  |  |  |
| **Mental Disorders due to alcohol (F10)** | **18** | Reference | ******* | ******* | ******* | 0.77 | 0.1 | 5.97 | 0.76 | 0.11 | 5.26 | 1.41 | 0.17 | 11.43 |
| **Alcoholic cardiomyopathy (I42.6)** | **121** | Reference | 1.27 | 0.46 | 3.52 | 1.09 | 0.5 | 2.38 | 1.11 | 0.53 | 2.3 | 1.53 | 0.68 | 3.46 |
| **Alcoholic liver disease (K70)** | **74** | Reference | 3.49 | 0.94 | 12.94 | 1.91 | 0.59 | 6.2 | 1.35 | 0.41 | 4.4 | 0.99 | 0.25 | 3.84 |
| **Acute Alcohol poisoning (X45)** | **95** | Reference | 0.25 | 0.04 | 1.45 | 0.86 | 0.41 | 1.8 | 0.89 | 0.44 | 1.77 | 0.96 | 0.41 | 2.23 |
| ***Overall*** | **308** | Reference | 1.18 | 0.58 | 2.42 | 1.51 | 0.9 | 2.56 | 1.28 | 0.77 | 2.12 | 1.5 | 0.84 | 2.69 |
| **External causes***:** |  |  |  |  |  |  |  |  |  |  |  |  |  |  |
| **Transport injuries (V01-V99)** | **42** | Reference | 1.21 | 0.33 | 4.5 | 2.05 | 0.74 | 5.65 | 1.7 | 0.62 | 4.61 | 1.77 | 0.51 | 6.18 |
| **Other accidental poisoning (X40-X49 except X45)** | **33** | Reference | 3.75 | 0.45 | 31.32 | 4.03 | 0.66 | 24.83 | 1.97 | 0.31 | 12.76 | 4.12 | 0.57 | 29.7 |
| **Drowning (W65-W74)** | **18** | Reference | 1.18 | 0.15 | 9.37 | 0.4 | 0.07 | 2.38 | 1.45 | 0.37 | 5.75 | 0.36 | 0.04 | 3.3 |
| **Exposure to cold (X31)** | **29** | Reference | ******* | ******* | ******* | 1.06 | 0.15 | 7.42 | 3.2 | 0.54 | 19.04 | 1.98 | 0.26 | 15.01 |
| **Other accidental deaths (V0-X59, no including V01-V99, X40-49, W65-W74, and X31)** | **35** | Reference | 1.88 | 0.18 | 19.87 | 2.29 | 0.35 | 15.18 | 5.77 | 1 | 33.26 | 1.41 | 0.16 | 12.44 |
| **Suicide (X60-X84)** | **120** | Reference | 0.49 | 0.12 | 1.91 | 1.84 | 0.84 | 4.03 | 2.10 | 0.98 | 4.49 | 2.85 | 1.25 | 6.52 |
| **Homicide (X85-Y09)** | **45** | Reference | ******* | ******* | ******* | 2.82 | 0.44 | 17.95 | 3.34 | 0.54 | 20.54 | 7.91 | 1.20 | 51.95 |
| **Undetermined intent (Y10-Y34)** | **111** | Reference | 2.27 | 0.85 | 6.05 | 1.56 | 0.68 | 3.59 | 1.43 | 0.63 | 3.24 | 1.92 | 0.77 | 4.78 |
| **All other external causes** | **16** | Reference | 0.88 | 0.11 | 7.18 | 0.31 | 0.05 | 2.11 | 0.69 | 0.15 | 3.25 | 0.62 | 0.06 | 6.15 |
| **Undetermined intent plus suicide****** | **231** | Reference | 1.15 | 0.53 | 2.50 | 1.64 | 0.91 | 2.94 | 1.75 | 0.99 | 3.08 | 2.44 | 1.30 | 4.56 |
| **All causes except external causes** | **1206** | Reference | 1.66 | 1.18 | 2.34 | 1.93 | 1.45 | 2.57 | 1.84 | 1.40 | 2.42 | 2.16 | 1.57 | 2.99 |
| *Adjusted for age + employment + car/central heating ownership + education + imprisonment + any adverse life event + marital status + alcohol-related dysfunction (zapoi, surrogates and acute alcohol-related dysfunction (latent)) +physical assault | | | | | | | | | | | | | | |

| **Table S2: Table showing the association between smoking and physical assault in controls adjusted for other potential confounding variables** | | | |
| --- | --- | --- | --- |
|  | **Adjusted* Odds Ratio for assault** | **Lower 95% CI** | **Upper 95% CI** |
| **Never smoker** | Reference | - | - |
| **Ex-Smoker** | 0.599 | 0.332 | 1.082 |
| **1-10/day current** | 1.133 | 0.77 | 1.666 |
| **11-20/day current** | 1.219 | 0.844 | 1.761 |
| **>20/day current** | 1.258 | 0.832 | 1.902 |

*Adjusted for age + employment + car/central heating ownership + education + imprisonment + any adverse life event + marital status + alcohol-related dysfunction (zapoi, surrogates and acute alcohol-related dysfunction (latent))

| **Table S3: Regression models for the association between mortality and physical assault with and without adjustment for smoking.** | | | | | | | |
| --- | --- | --- | --- | --- | --- | --- | --- |
|  |  | **Model II without smoking: adjusted for age, car/central heating ownership, education, imprisonment, adverse life event, marital status** | | | **Model II with smoking: adjusted for age, smoking, car/central heating ownership, education, imprisonment, adverse life event, marital status** | | |
| **Chapter-level causes (ICD-10 codes):** | **Number of cases** | **Odds ratio** | **Lower 95% CI** | **Upper 95% CI** | **Odds ratio** | **Lower 95% CI** | **Upper 95% CI** |
| **Infections and parasitic diseases (I)** | **53** | **1.21** | 0.47 | 3.12 | **1.29** | 0.29 | 5.77 |
| **Neoplasms (II)** | **170** | **1.61** | 0.87 | 2.99 | **1.58** | 0.59 | 4.24 |
| **Mental and behavioural disorders (V)** | **19** | **3.3** | 1.17 | 9.29 | **3.02** | 0.6 | 15.31 |
| **Diseases of circulatory system (IX)** | **573** | **2.26** | 1.63 | 3.14 | **2.09** | 1.25 | 3.51 |
| **Diseases of respiratory system (X)** | **137** | **1.71** | 0.96 | 3.03 | **1.67** | 0.68 | 4.09 |
| **Diseases of digestive system (XI)** | **182** | **2.93** | 1.88 | 4.58 | **2.76** | 1.37 | 5.55 |
| **External causes (XX)** | **544** | **4.15** | 3.11 | 5.53 | **3.82** | 2.43 | 6 |
| **Other** | **72** | **2.05** | 1.01 | 4.14 | **2.2** | 6.77 | 0.72 |
| ***All causes of death*** | ***1750*** | ***3.09*** | *2.43* | *3.93* | ***2.92*** | *2* | *4.26* |
| **Selected causes:** |  |  |  |  |  |  |  |
| **Circulatory disease**:** |  |  |  |  |  |  |  |
| **Ischaemic Heart Disease (I20-25)** | **258** | **1.48** | 0.9 | 2.44 | **1.3** | 0.6 | 2.85 |
| **Other Cardiomyopathy (I42, except I42.6)** | **61** | **1.05** | 0.44 | 2.49 | **0.88** | 0.22 | 3.45 |
| **Cerebrovascular disease (I60-69)** | **100** | **3.27** | 1.81 | 5.9 | **2.92** | 1.15 | 7.41 |
| **Other circulatory disease (I00-I99, except I20-25, I42, and I60-69)** | **33** | **1.04** | 0.34 | 3.21 | **1.03** | 0.17 | 6.04 |
| **Alcohol-related:** |  |  |  |  |  |  |  |
| **Mental Disorders due to alcohol (F10)** | **18** | **3.59** | 1.26 | 10.26 | **3.12** | 0.61 | 16.04 |
| **Alcoholic cardiomyopathy (I42.6)** | **121** | **4.08** | 2.54 | 6.57 | **3.74** | 1.77 | 7.91 |
| **Alcoholic liver disease (K70)** | **74** | **4.66** | 2.6 | 8.34 | **4.65** | 1.84 | 11.75 |
| **Acute Alcohol poisoning (X45)** | **95** | **3.94** | 2.34 | 6.34 | **3.57** | 1.58 | 8.09 |
| ***Overall*** | **308** | ***4.51*** | *3.18* | *6.4* | ***4.19*** | *2.42* | *7.26* |
| **External causes***:** |  |  |  |  |  |  |  |
| **Transport injuries (V01-V99)** | **42** | **4.22** | 1.99 | 8.94 | **4.18** | 1.27 | 13.74 |
| **Other accidental poisoning (X40-X49 except X45)** | **33** | **1.5** | 0.62 | 3.64 | **1.47** | 0.35 | 6.12 |
| **Drowning (W65-W74)** | **18** | **0.55** | 0.09 | 3.36 | **0.46** | 0.03 | 7.89 |
| **Exposure to cold (X31)** | **29** | **5.8** | 2.59 | 12.96 | **5.15** | 1.48 | 17.95 |
| **Other accidental deaths (V0-X59, no including V01-V99, X40-49, W65-W74, and X31)** | **35** | **2.09** | 0.82 | 5.38 | **1.83** | 0.42 | 8.01 |
| **Suicide (X60-X84)** | **120** | **3.96** | 2.51 | 6.24 | **3.36** | 1.64 | 6.89 |
| **Homicide (X85-Y09)** | **45** | **8.66** | 4.32 | 16.36 | **7.68** | 2.6 | 22.71 |
| **Undetermined intent (Y10-Y34)** | **111** | **4.67** | 2.89 | 7.55 | **4.39** | 2.07 | 9.32 |
| **All other external causes** | **16** | **0.91** | 0.15 | 5.72 | **0.87** | 0.05 | 15.91 |
| **Undetermined intent plus suicide****** | **231** | **4.44** | 3.09 | 6.38 | **4.28** | 4.03 | 7.13 |
| **All causes except external causes** | **1206** | **2.42** | 1.84 | 3.19 | **2.32** | 1.51 | 3.58 |

| **Table S4: Regression models for the association between physical assault and mortality, with and without adjustment for socioeconomic position.** | | | | | | | | | | | | | |
| --- | --- | --- | --- | --- | --- | --- | --- | --- | --- | --- | --- | --- | --- |
|  |  | **Model II without SEP: age, smoking, imprisonment, adverse life event, marital status** | | | **Model II: age, smoking, car/central heating ownership, education, employment, imprisonment, adverse life event, marital status** | | | **Model IV without socio-economic position: age, smoking, imprisonment, adverse life event, marital status, alcohol-related dysfunction** | | | **Model IV: age, smoking, car/central heating ownership, education, employment, imprisonment, adverse life event, marital status, alcohol-related dysfunction** | | |
| **Chapter-level causes (ICD-10 codes):** | **Number of cases** | **Odds ratio** | **Lower 95% CI** | **Upper 95% CI** | **Odds ratio** | **Lower 95% CI** | **Upper 95% CI** | **Odds ratio** | **Lower 95% CI** | **Upper 95% CI** | **Odds Ratio** | **Lower 95% CI** | **Upper 95% CI** |
| **Infections and parasitic diseases (I)** | **53** | **1.55** | 0.63 | 3.8 | **1.29** | 0.29 | 5.77 | **0.88** | 0.34 | 2.31 | **1.33** | 0.36 | 5.01 |
| **Neoplasms (II)** | **170** | **1.89** | 1.06 | 3.4 | **1.58** | 0.59 | 4.24 | **1.66** | 0.92 | 3.01 | **1.66** | 0.67 | 4.12 |
| **Mental and behavioural disorders (V)** | **19** | **3.69** | 1.37 | 9.91 | **3.02** | 0.6 | 15.31 | **1.86** | 0.58 | 5.96 | **2.22** | 0.42 | 11.76 |
| **Diseases of circulatory system (IX)** | **573** | **2.43** | 1.79 | 3.32 | **2.09** | 1.25 | 3.51 | **1.86** | 1.33 | 2.6 | **2.07** | 1.24 | 3.46 |
| **Diseases of respiratory system (X)** | **137** | **2.21** | 1.29 | 3.78 | **1.67** | 0.68 | 4.09 | **1.23** | 0.7 | 2.35 | **1.38** | 0.56 | 3.36 |
| **Diseases of digestive system (XI)** | **182** | **3.33** | 2.18 | 5.07 | **2.76** | 1.37 | 5.55 | **1.85** | 1.11 | 3.08 | **1.78** | 0.83 | 3.78 |
| **External causes (XX)** | **544** | **4.09** | 3.11 | 5.38 | **3.82** | 2.43 | 6 | **3.07** | 2.26 | 4.16 | **3.39** | 2.12 | 5.4 |
| **Other** | **72** | **2.81** | 1.43 | 5.52 | **2.2** | 6.77 | 0.72 | **2.1** | 1 | 4.41 | **2** | 0.68 | 5.84 |
| ***All causes of death*** | ***1750*** | ***3.21*** | *2.55* | *4.03* | ***2.92*** | *2* | *4.26* | ***2.32*** | *1.81* | *2.96* | ***2.42*** | *1.63* | *3.59* |
| **Selected causes:** |  |  |  |  |  |  |  |  |  |  |  |  |  |
| **Circulatory disease**:** |  |  |  |  |  |  |  |  |  |  |  |  |  |
| **Ischaemic Heart Disease (I20-25)** | **258** | **1.47** | 0.91 | 2.37 | **1.3** | 0.6 | 2.85 | **1.3** | 0.79 | 2.13 | **1.52** | 0.71 | 3.24 |
| **Other Cardiomyopathy (I42, except I42.6)** | **61** | **1.15** | 0.49 | 2.71 | **0.88** | 0.22 | 3.45 | **0.74** | 0.3 | 1.84 | **0.93** | 0.24 | 3.63 |
| **Cerebrovascular disease (I60-69)** | **100** | **3.17** | 1.8 | 5.58 | **2.92** | 1.15 | 7.41 | **2.65** | 1.46 | 4.81 | **3.08** | 1.27 | 7.46 |
| **Other circulatory disease (I00-I99, except I20-25, I42, and I60-69)** | **33** | **1.5** | 0.51 | 4.4 | **1.03** | 0.17 | 6.04 | **1.31** | 0.43 | 3.96 | **1.68** | 0.31 | 9.16 |
| **Alcohol-related:** |  |  |  |  |  |  |  |  |  |  |  |  |  |
| **Mental Disorders due to alcohol (F10)** | **18** | **3.9** | 1.44 | 10.56 | **3.12** | 0.61 | 16.04 | **1.9** | 0.58 | 6.16 | **2.37** | 0.43 | 13.01 |
| **Alcoholic cardiomyopathy (I42.6)** | **121** | **4.41** | 2.85 | 6.85 | **3.74** | 1.77 | 7.91 | **2.62** | 1.52 | 4.52 | **2.87** | 1.25 | 6.57 |
| **Alcoholic liver disease (K70)** | **74** | **5.51** | 3.14 | 9.67 | **4.65** | 1.84 | 11.75 | **2.82** | 1.4 | 5.68 | **2.69** | 1.02 | 7.12 |
| **Acute Alcohol poisoning (X45)** | **95** | **3.9** | 2.39 | 6.37 | **3.57** | 1.58 | 8.09 | **2.06** | 1.14 | 3.69 | **2.44** | 1.01 | 5.92 |
| ***Overall*** | **308** | ***4.43*** | *3.22* | *6.1* | ***4.19*** | *2.42* | *7.26* | ***2.58*** | *1.71* | *3.88* | ***2.82*** | *1.52* | *5.26* |
| **External causes***:** |  |  |  |  |  |  |  |  |  |  |  |  |  |
| **Transport injuries (V01-V99)** | **42** | **3.89** | 1.86 | 8.13 | **4.18** | 1.27 | 13.74 | **4.61** | 2.11 | 10.07 | **4.79** | 1.52 | 15.14 |
| **Other accidental poisoning (X40-X49 except X45)** | **33** | **1.3** | 0.66 | 4.02 | **1.47** | 0.35 | 6.12 | **1.22** | 0.47 | 3.15 | **1.92** | 0.51 | 7.31 |
| **Drowning (W65-W74)** | **18** | **0.57** | 0.09 | 3.46 | **0.46** | 0.03 | 7.89 | **0.28** | 0.04 | 1.96 | **0.27** | 0.01 | 5.1 |
| **Exposure to cold (X31)** | **29** | **5.55** | 2.52 | 12.23 | **5.15** | 1.48 | 17.95 | **3.6** | 1.54 | 8.43 | **4.43** | 1.25 | 15.67 |
| **Other accidental deaths (V0-X59, no including V01-V99, X40-49, W65-W74, and X31)** | **35** | **2.36** | 0.96 | 5.82 | **1.83** | 0.42 | 8.01 | **1.44** | 0.52 | 4.04 | **1.34** | 0.3 | 6.06 |
| **Suicide (X60-X84)** | **120** | **3.62** | 2.33 | 5.61 | **3.36** | 1.64 | 6.89 | **2.66** | 1.66 | 4.26 | **2.96** | 1.44 | 6.09 |
| **Homicide (X85-Y09)** | **45** | **8.68** | 4.41 | 17.08 | **7.68** | 2.6 | 22.71 | **6.76** | 3.29 | 13.89 | **5.93** | 2.07 | 17 |
| **Undetermined intent (Y10-Y34)** | **111** | **5.13** | 3.22 | 8.16 | **4.39** | 2.07 | 9.32 | **3.4** | 2.03 | 5.69 | **3.43** | 1.59 | 7.38 |
| **All other external causes** | **16** | **1.11** | 0.19 | 6.64 | **0.87** | 0.05 | 15.91 | **0.58** | 0.08 | 4.06 | **0.58** | 0.03 | 10.61 |
| **Undetermined intent plus suicide****** | **231** | **4.44** | 3.14 | 6.39 | **2.28** | 4.03 | 7.13 | **3.18** | 2.17 | 4.67 | **3.39** | 1.9 | 6.07 |
| **All causes except external causes** | **1206** | **2.7** | 20.09 | 3.48 | **2.32** | 1.51 | 3.58 | **1.88** | 1.42 | 2.49 | **1.97** | 1.28 | 3.02 |
